# Changing Incidence of Invasive Pneumococcal Disease in Infants Less Than 90 Days of Age Before and After Introduction of the 13-Valent Pneumococcal Conjugate Vaccine in Blantyre, Malawi: A 14-Year Hospital Based Surveillance Study

**DOI:** 10.1097/INF.0000000000003606

**Published:** 2022-06-13

**Authors:** Marianne Koenraads, Todd D. Swarthout, Naor Bar-Zeev, Comfort Brown, Jacquline Msefula, Brigitte Denis, Queen Dube, Stephen B. Gordon, Robert S. Heyderman, Melissa J. Gladstone, Neil French

**Affiliations:** From the * Department of Women and Children’s Health, Institute of Life Course and Medical Sciences, University of Liverpool, Liverpool, UK; † Malawi-Liverpool-Wellcome Trust Clinical Research Programme, Kamuzu University of Health Sciences, Blantyre, Malawi; ‡ NIHR Global Health Research Unit on Mucosal Pathogens, Research Department of Infection, Division of Infection and Immunity, University College London, London, United Kingdom; § Department of Clinical Infection, Microbiology and Immunology, Institute of Infection Veterinary and Ecological Science, University of Liverpool, Liverpool, United Kingdom; ¶ International Vaccine Access Center, Department of International Health, Bloomberg School of Public Health, Johns Hopkins University, Baltimore, MD, United States; ‖ Department of Paediatrics, Kamuzu University of Health Sciences, Blantyre, Malawi; ** Liverpool School of Tropical Medicine, Liverpool, United Kingdom.

**Keywords:** invasive pneumococcal disease, infant, pneumococcal conjugate vaccine

## Abstract

**Background:**

Invasive pneumococcal disease (IPD) in young infants is uncommon but associated with high morbidity and mortality. Accurate data on the burden of IPD in young infants in low-income countries are lacking. We examined the burden of IPD in infants <90 days old in Blantyre, Malawi over a 14-year period and evaluated the indirect impact of the 13-valent pneumococcal conjugate vaccine (PCV13) on vaccine-serotype IPD (VT-IPD) in this population.

**Methods:**

We conducted laboratory-based prospective IPD surveillance in infants <90 days of age admitted to Queen Elizabeth Central Hospital in Blantyre between 2005 and 2018, including 7 years pre-PCV13 and 7 years post-PCV13 introduction. IPD was defined as *Streptococcus pneumoniae* identified by culture from blood or cerebrospinal fluid. Serotypes were determined by multiplex polymerase chain reaction and latex agglutination testing.

**Results:**

We identified 130 cases of culture-confirmed IPD in infants <90 days old between 2005 and 2018. Total IPD incidence was declining before PCV13 introduction. The mean incidence of IPD was significantly lower in the post-PCV13 era. Serotypes 5 (27.8%) and 1 (15.6%) were most prevalent. Even after PCV13 introduction, VTs remained the primary cause of IPD, with serotype 5 accounting for 17.4% and serotype 1 for 13.0% of cases in young infants.

**Conclusion:**

Vaccine serotypes 1 and 5 were the main cause of IPD in neonates and young infants, both before and after PCV13 introduction. This suggests incomplete indirect protection with persisting VT carriage across the population despite vaccination in this setting. Alternative vaccine schedules and other vaccine introduction approaches need to be considered to protect this vulnerable population.

## BACKGROUND

*Streptococcus pneumoniae* is a major cause of serious bacterial infections, including pneumonia, sepsis and meningitis in young children. Globally, there were an estimated 294,000 pneumococcal deaths in HIV-uninfected children 1–59 months old in 2015, with the majority occurring in sub-Saharan Africa and Asia.^1^ *S. pneumoniae* is considered an uncommon but well-recognized cause of invasive bacterial disease in neonates and young infants and has been associated with high morbidity and mortality, with a case fatality rate of up to 14.3%.^2–6^ The global burden of neonatal invasive pneumococcal disease (IPD) has been estimated at 36.0 per 100,000 live births in the pre-pneumococcal conjugate vaccine (PCV) period.^7^ However, accurate data on the burden of IPD in neonates and young infants are lacking, especially in low-income countries.

In Malawi, the under-5 child mortality rate was reduced by two-thirds between 1990 and 2015, with the country, therefore, achieving Millennium Development Goal (MDG) 4. The neonatal mortality declined more slowly (from 50 to 23 deaths per 1000 live births) and remains among the highest in the world.^8,9^ Severe bacterial infections contribute significantly as a leading cause of death in the neonatal population.^10^ Work examining the etiology of neonatal sepsis in Blantyre, Malawi from 1996 to 2001 showed that *S. pneumoniae* was responsible for 10% of neonatal sepsis cases and 23% of neonatal meningitis cases.^11^

Supported by Gavi, the Vaccine Alliance, the Vaccine Alliance, the 13-valent PCV (PCV13) was introduced in Malawi in November 2011 as part of the national expanded program of immunization with a 3 + 0 schedule (one dose at 6, 10 and 14 weeks of age). A recent study by Swarthout et al^12^ has shown that 7 years after PCV13 introduction in Malawi, despite high vaccine uptake and a reduction in vaccine-type (VT) carriage, there remains high persistent residual carriage. In this study, we evaluated the burden of IPD in infants <90 days old admitted to Queen Elizabeth Central Hospital (QECH) in Blantyre, Malawi in the pre- and post-PCV13 period.

## METHODS

### Study Setting

Malawi is a landlocked country in southern sub-Saharan Africa with a population of 19.1 million people. The country is ranked by the World Bank in the lowest income category.^13^ Located in Blantyre, the capital city of Malawi’s southern region, QECH is a large government-funded district and referral hospital with about 25,000 pediatric admissions a year. QECH provides free medical care to the 1.3 million urban, peri-urban and rural residents of Blantyre District.

### Case Ascertainment and Laboratory Confirmation

In accordance with longstanding clinical guidelines, all young infants presenting to QECH with fever (axillary temperature >37·5°C) or clinical suspicion of sepsis or meningitis undergo blood cultures and, where appropriate, lumbar puncture. We have been conducting sentinel surveillance for laboratory-confirmed bloodstream infection and meningitis (including IPD) in all age groups at QECH since 1998, as previously described.^14–16^

Specimens were processed at the co-located Malawi-Liverpool-Wellcome Clinical Research Programme laboratory, using BD BACTEC (Becton Dickinson, Franklin Lakes, NJ). Those positive by BACTEC were Gram stained. Gram-positive diplococci or Gram-positive cocci in short chains were initially classified as *S. pneumoniae* after testing negative using the catalase test. These isolates were archived on Microbank beads (ProLab Diagnostics) at −80^o^C. For subsequent confirmation of *S. pneumoniae* and serotyping, archived isolates were plated on gentamicin-sheep blood agar (SBG; 7% sheep blood, 5 µL gentamicin/mL) and incubated overnight at 37°C in 5% CO_2_. *S. pneumoniae* growth was confirmed by colony morphology and optochin disc (Oxoid, Basingstoke, UK) susceptibility. The bile solubility test was used on isolates with no or intermediate (zone diameter <14 mm) optochin susceptibility. A single colony of confirmed pneumococcus was selected and grown on a new SBG plate as before. Growth from this second plate was used for serotyping by latex agglutination (ImmuLex 7-10-13-valent Pneumotest; Statens Serum Institute, Denmark). The ImmuLex kit allows for differential identification of each PCV13 VT but not for differential identification of non-VT (NVT) serotypes; all pneumococcal isolates that were not VT were therefore reported as NVT. Nucleic acid amplification-based serotyping was performed on samples collected between January 1, 2009 and December 31, 2013, using the ‘Triplex sequential real-time polymerase chain reaction-serotyping Africa’ protocol of the Centers for Disease Control and Prevention.^17^ Both assays have been shown to be highly accurate and concordant in pneumococcal serotyping.^18,19^ There was 100% concordance among a random selection (approximately 6%) of serotyped isolates sent for confirmatory serotyping by Quellung reaction at the regional pneumococcal reference laboratory at the National Institute for Communicable Disease in Johannesburg, South Africa. Since 13 August 2011, serotyping has occurred in real time with specimen processing. Isolates collected before 13 August 2011 were retrospectively serotyped. Demographic information (including age and sex) was collected at the time of sampling. Clinical data were not available for prospective collection.

### Case Definitions

We analyzed all archived pneumococcal isolates from blood and cerebrospinal fluid (CSF) of infants less than 90 days old admitted to QECH between January 1, 2005 and December 31, 2018. IPD cases were defined as isolation of *S. pneumoniae* from a normally sterile site (ie, blood or CSF). We defined those with a positive CSF culture as “meningitis” and those with a positive blood culture as “bacteremia”. Cases with both a positive CSF and a positive blood culture were classified as “meningitis”. Although it is standard practice to take a CSF sample in all young infants with suspected sepsis, this was not done in all individuals. Cases in infants ≤7 days old were defined as “early-onset” disease and cases in infants 8–89 days old as “late-onset” disease.

### Statistical Analysis

We used descriptive statistics for demographic and clinical characteristics. Incidence rates were calculated using the annual number of VT, NVT and total (VT+NVT) IPD cases in infants <90 days old multiplied by 100,000 and this was divided by the annual age-specific population estimates for Blantyre. Population estimates were obtained from the 1998 and 2018 National Population Projections by Malawi’s National Statistical Office.^20,21^ We used linear interpolation of the intercensal period to estimate the year-by-year population estimates of children <1-year-old and estimated the population of infants <90-days-old by taking a proportion (3/12) of this. Confidence intervals (CIs) were estimated using the modified Wald method. Incidence rate ratios (IRRs) for the invasive pneumococcal disease were calculated over the study duration by log-binomial regression using years (365.25 days) between study start and PCV13 introduction (2011) and from PCV13 introduction to the end of the study period, coded as a single time variable.

## RESULTS

We identified a total of 130 cases of confirmed IPD in infants <90 days old over the study’s 14-year period, January 1, 2005–December 31, 2018. The median age at hospital presentation was 30 days. Among the 130 infant cases, 93 (71.5%) presented with meningitis and 36 (27.7%) presented with early-onset disease (0–7 days old); among which 21 (58.3%) were meningitis and 15 (41.7%) were bacteremia. Among the 94 (72.3%) infants with late-onset disease (8–89 days old), 72 (76.6%) were meningitis and 22 (23.4%) were bacteremia. A total of 104 cases of IPD occurred before the November 2011 introduction of PCV13. Twenty-five (24.0%) of these were early-onset and 79 (76.0%) late-onset disease. Among the 26 IPD cases in the post-PCV13 era, 11 (42.3%) were early-onset and 15 (57.7%) late-onset disease (Table 1). Of the 36 total early-onset cases, 17 (47.2%) presented in the first 72 hours of life and 19 (52.8%) on days 4–7.

**TABLE 1.** Early vs Late-Onset Disease in the Pre- and Post-PCV13 Periods

Clinical Syndrome	Total		Pre-PCV13		Post-PCV13	
	n	(%)	n	(%)	n	(%)
Total	130		104		26	
Meningitis	93	(71.5)	74	(71.2)	19	(73.1)
Bacteremia	37	(28.5)	30	(28.8)	7	(26.9)
Early onset (0–7 days)	36	(27.7)	25	(24.0)	11	(42.3)
Meningitis	21	(58.3)	13	(52.0)	8	(72.7)
Bacteremia	15	(41.7)	12	(48.0)	3	(27.3)
Late onset (8–89 days)	94	(72.3)	79	(76.0)	15	(57.7)
Meningitis	72	(76.6)	61	(77.2)	11	(73.3)
Bacteremia	22	(23.4)	18	(22.8)	4	(26.7)

Pre-PCV13 period, January 1, 2005–November 11, 2011.

Post-PCV13 period January1, 2012–December 31, 2018.

PCV indicates pneumococcal conjugate vaccine.

### Serotype Distribution

Among the total 130 cases, we were able to recover and serotype 90 (69.2%) samples. Analysis of isolates that were and were not recoverable showed no statistically significant difference in age, gender or sample type (data not shown). Over the duration of the study period, vaccine serotypes 5 (27.8%) and 1 (15.6%) were the most commonly identified in this population, with NVT-IPD accounting for 32.2% of the total recovered samples. In the pre-PCV13 period, 70.1% of IPD cases were caused by VT and 29.9% by NVT. In the early post-PCV13 period (2012–2015) 56.3% were VT and 43.5% NVT and in the late post-PCV13 period (2016–2018) 57.1% were VT and 42.9% were NVT. The most frequent serotypes in the post-PCV13 period remained vaccine serotypes 5 and 1 (Table 2).

**TABLE 2.** Serotype Distribution in Infants <90 Days in the Pre-PCV and Post-PCV Periods

Serotype	Total IPD		2005–2011(Pre-PCV13)		2012–2015(Post-PCV13)		2016–2018(Post-PCV13)	
	n	(%)	n	(%)	n	(%)	n	(%)
1	14	(15.6)	11	(16.4)	3	(18.8)	0	(0.0)
3	2	(2.2)	2	(3.0)	0	(0.0)	0	(0.0)
4	1	(1.1)	1	(1.5)	0	(0.0)	0	(0.0)
5	25	(27.8)	21	(31.3)	2	(12.5)	2	(28.6)
6A/B	4	(4.4)	3	(4.5)	0	(0.0)	1	(14.3)
7F	5	(5.6)	4	(6.0)	1	(6.3)	0	(0.0)
9V	1	(1.1)	1	(1.5)	0	(0.0)	0	(0.0)
14	2	(2.2)	0	(0.0)	1	(6.3)	1	(14.3)
18C	3	(3.3)	3	(4.5)	0	(0.0)	0	(0.0)
19A	0	(0.0)	0	(0.0)	0	(0.0)	0	(0.0)
19F	0	(0.0)	0	(0.0)	0	(0.0)	0	(0.0)
23F	4	(4.4)	2	(3.0)	2	(12.5)	0	(0.0)
VT total	61	(67.8)	48	(71.6)	9	(56.2)	4	(57.1)
NVT total	29	(32.2)	19	(28.4)	7	(43.8)	3	(42.9)
Unrecovered	40	–	37	–	3	–	0	–
Total	130	–	104	–	19	–	7	–

PCV indicates pneumococcal conjugate vaccine; NVT, non-vaccine serotype; VT, vaccine serotype.

### IPD Incidence

**FIGURE 1.**
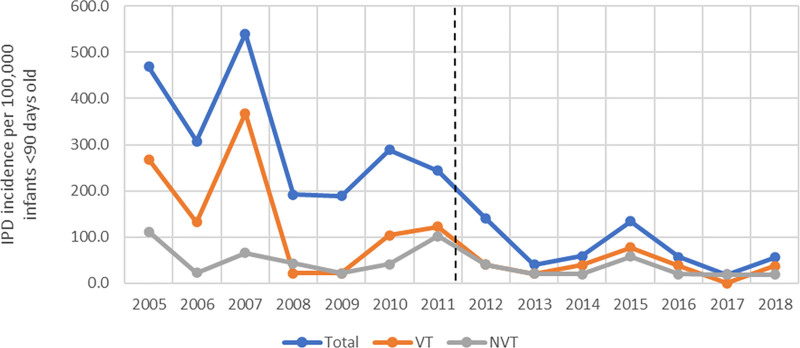
Incidence of invasive pneumococcal disease (IPD) in infants <90 days old (per 100,000 children <90 days old) in Blantyre, 2005–2018. NVT indicates non-vaccine type; VT, vaccine type. Dashed line indicates date of PCV13 introduction. Refer to Table 1, Supplemental Digital Content, http://links.lww.com/INF/E756 for incidence per year.

We estimated the annual IPD incidence rates per 100,000 infants <90 days old in Blantyre over the 14-year period. As reported elsewhere for all-age IPD,^14^ IPD incidence was already declining before the 2011 introduction of PCV13. After the introduction of PCV13, there was a further decline in IPD cases in young infants (Figure 1, Supplemental Digital Content, http://links.lww.com/INF/E756).

The mean annual incidence of total (VT+NVT) IPD in infants <90 days old in the pre-PCV13 period was 319 (95% CI: 264–385) per 100,000 versus 72 (49–106) per 100,000 in the post-PCV13 period (*P* < 0.01). For VT-IPD, the mean incidence pre-PCV13 was 148 (116–179) per 100,000 versus 36 (18–57) per 100,000 infants <90 days old in the post-PCV13 period. And for NVT-IPD the mean incidence pre-PCV13 was 58 (35–84) per 100,000 infants <90 days old versus 27 (11–49) per 100,000 infants <90 days old in the post-PCV13 period. The overall reduction in total IPD incidence among infants <90 days of age from 2005 to 2018 was 12% per year (IRR: 0.88; 95% CI: 0.86–0.90; *P*<0·001). For the years following the PCV13 introduction (2012–2018) there was a significant and further reduction of 46% (IRR 0.54, 0.46, 0.64; *P* < 0.001).

## DISCUSSION

In this low-income sub-Saharan African population with a high burden of disease, in which invasive pneumococcal disease incidence was already decreasing, we used robust long-term hospital-based surveillance to show a continued reduction in the incidence of VT-IPD among children <90 days of age following the introduction of PCV13. However, our results also show that *S. pneumoniae* remains an important pathogen in causing bacteremia and meningitis in neonates and young infants in Blantyre, Malawi.

The 7-valent pneumococcal conjugate vaccine was first introduced in the United States (US) in 2000 and resulted in a 76% decline in overall IPD incidence for children <5 years.^22^ Several studies in other high-income settings have also reported significant reductions in IPD in neonates and young PCV-unvaccinated infants after the introduction of PCV, suggesting protection through both direct and indirect effects. Ladhani et al^23^ showed that in England and Wales introduction of PCV7 was responsible for an 83% reduction in VT-IPD in infants <90 days old and a declining trend in overall IPD. Similarly, studies in the US among neonates reported a 40%–74% reduction in IPD after PCV7 introduction.^24,25^

Our study shows that in Blantyre, the incidence of IPD in young infants was already declining before PCV13 introduction on 12 November 2011, comparable to the trend of decreasing IPD reported by Bar-Zeev et al across all age groups in Blantyre.^14^ Similar findings were also described in a longitudinal household study of pneumococcal acquisition among infants exposed to HIV in Karonga, northern Malawi,^26^ and in a study on pediatric bloodstream infections in Malawi.^27^ Possible explanations for this pre-PCV13 secular decline in IPD include the down-stroke of long-term cyclical change, improved food security and nutrition in this population and improved HIV care. From 2004 to 2015, the number of new patients started on antiretroviral therapy in Malawi increased from about 3000 to over 820,000, likely having a positive impact on the health of mothers and thereby improved infant wellbeing.^28^ Following the introduction of PCV13, IPD incidence in neonates and young infants in Blantyre further declined, consistent with an indirect effect of vaccination. However, it is not possible to disassociate the contribution of PCV13 from the preexisting secular trend.

Nonetheless, evidence is clear that VT-IPD remains present in this population, up to 7 years after PCV13 introduction. In our study, more than 50% of cases in neonates and young infants in the post-PCV period were due to VT, with serotypes 1 and 5 being most common both before and after PCV13 introduction. In sub-Saharan Africa, rates of pneumococcal carriage, a prerequisite for disease, are reported to be high with carriage rates of up to 90% described in the pre-PCV period.^29–31^ There is increasing evidence that in this setting the current strategy used to implement conjugate vaccines does not achieve the optimal reductions in VT pneumococcal carriage as seen in resource-rich countries. Recently, it has been shown that in Malawi, despite high PCV13 uptake, there remains a high persistent residual carriage of all PCV13 serotypes.^12^ Serotype 1, a common cause of IPD in Africa,^32^ was responsible for 3% of VT carriage of all ages in that study, consistent with our present findings in infants in this population. Furthermore, another study in northern Malawi demonstrated a reduction in VT carriage after PCV13 introduction but found high carriage rates continued to be present among age-ineligible (6-weeks old) infants, with no difference in pneumococcal acquisition between the pre- and post-PCV13 period.^31^ The limited vaccine impact on the carriage in this setting is likely largely driven by demographic and socio-economic factors, and a local higher, age-dependent force of infection.^33^

The majority (72%) of IPD cases in our study were of late-onset disease, which is likely due to acquisition within households.^34,35^ Previous studies in low-income settings have described that pneumococcal acquisition occurs very early in life. Tigoi et al^36^ showed that in Kilifi, Kenya the median time to acquisition was 38.5 days of life and Heinsbroek et al^26^ showed a median time to first acquisition of 59 days of life in northern Malawi.

In our study, 36 (27.7%) of our cases occurred in the first 7 days of life with 17 of these (47.2%) occurring within the first 72 hours after birth. Although horizontal transmission from the mother or other household members remains likely, this suggests potential perinatal transmission at the time of labor. Although *S. pneumoniae* is rarely isolated from the female genital tract, it is responsible for 1%–11% of cases of neonatal sepsis.^11,37,38^ This suggests that mothers colonized with *S. pneumoniae* in the genital tract have a high likelihood of transmitting the organism to their infants at or very soon after birth.^2,3,39^ Our results emphasize the need to prevent mother-to-child transmission and the importance of further research into preventative strategies such as maternal immunization.

Although this work provides an estimate of vaccine impact, it has several limitations. We were unable to review clinical, follow-up and outcome data for the cases and were not able to collect information on demographics and vaccination status. Our study only represents children who presented to the hospital and not those managed in community health centers or at home, with a resulting risk of underestimation of true numbers. This is likely limited, given the clinical severity of IPD in infants and the fact that QECH is the only hospital in Blantyre District with inpatient pediatric facilities. There remains a further risk of underestimation of IPD due to the challenges with blood volumes for culture in small infants. To our benefit, a large longitudinal study on bloodstream infections in children admitted to QECH has shown that the total pediatric and neonatal admissions have remained broadly constant since 2005.^25^ Furthermore, we estimated the population of infants <90 days old by taking a 3/12 proportion of the children <1-year-old. This method will lead to a small underestimation of the denominator due to the high infant mortality rates (IMR). This underestimation will change over time as a consequence of falling IMR. Although the fall in IMR is relatively substantial from about 70 to 40 per 1000 live births, in absolute terms this makes only marginal differences to the denominator population and we feel is an acceptable error in the context of these data. Though not all isolates were recoverable, analysis of those that were and were not recoverable showed no statistically significant difference in age, gender or sample type.

This study presents a unique set of data on the burden of IPD in infants less than 90 days old over a 14-year period. The results are derived from one of the few large databases across Africa based on long-term robust continuous prospective, systematic and reproducible surveillance. The Malawi-Liverpool-Wellcome Clinical Research Programme has provided routine, quality controlled, diagnostic blood culture service for pediatric patients with suspected severe bacterial infection admitted to QECH since 1998. Previously published robust surveillance studies at this site have shown similar methodology and consistency.^15,27^

## CONCLUSION

This study demonstrates that IPD incidence among neonates and young infants has declined over the past decade in Blantyre, Malawi. However, pneumococcal vaccine serotypes were the main cause of IPD both before and after PCV13 introduction. We believe that there is incomplete indirect protection in this group, of which most are too young to derive direct protection from vaccination. Strategies such as maternal or neonatal immunization or schedule change with a booster dose to achieve greater reductions in the general population carriage need to be considered to protect this vulnerable population. Further studies to evaluate schedule change in this setting are underway.

## ACKNOWLEDGMENTS

We thank the MLW laboratory management team (led by Brigitte Denis) and the MLW data management team (led by Clemens Masesa). Furthermore, we thank all the staff of the Chatinkha Neonatal Care Unit and the Paediatric Department at QECH for their efforts in caring for the most vulnerable infants and contributing to the collection of samples for this study.

## Supplementary Material


