# Supplementary material for: Changing Incidence of Invasive Pneumococcal Disease in Infants Less Than 90 Days of Age Before and After Introduction of the 13-Valent Pneumococcal Conjugate Vaccine in Blantyre, Malawi: A 14-Year Hospital Based Surveillance Study
Source: Pediatr Infect Dis J. 2022 Jun 13;41(9):764–8. doi: 10.1097/INF.0000000000003606 (PMC9359774; doi:10.1097/INF.0000000000003606)
Supplement: Supplementary file 1 [file inf-41-0764-s001.docx]

**Supplementary Material**

Marianne Koenraads et al.

Changing incidence of invasive pneumococcal disease in infants less than 90 days of age before and after introduction of the 13-valent Pneumococcal Conjugate Vaccine in Blantyre, Malawi: a 14-year hospital-based surveillance study

Table S1. IPD Incidence, per 100,000 population <90 days old, Blantyre Malawi

| **Year** | **Total** | **VT** | **NVT** |
| --- | --- | --- | --- |
| 2005 | 469.4 | 268.2 | 111.8 |
| 2006 | 307.8 | 131.9 | 22.0 |
| 2007 | 540.7 | 367.7 | 64.9 |
| 2008 | 191.6 | 21.3 | 42.6 |
| 2009 | 188.6 | 21.0 | 21.0 |
| 2010 | 288.8 | 103.1 | 41.3 |
| 2011 | 243.8 | 121.9 | 101.6 |
| 2012 | 140.1 | 40.0 | 40.0 |
| 2013 | 39.4 | 19.7 | 19.7 |
| 2014 | 58.3 | 38.9 | 19.4 |
| 2015 | 134.0 | 76.6 | 57.4 |
| 2016 | 56.6 | 37.8 | 18.9 |
| 2017 | 18.6 | 0.0 | 18.6 |
| 2018 | 55.1 | 36.7 | 18.4 |
